# Prospects for the development of community-based care in remote rural areas: a stakeholder analysis in Laos

**DOI:** 10.1186/s12913-023-10523-6

**Published:** 2024-01-11

**Authors:** Marco Liverani, Khampheng Phongluxa, Koukeo Phommasone, Rusheng Chew, Arjun Chandna, Tiengkham Pongvongsa, Mayfong Mayxay, Sengchanh Kounnavong, Elizabeth Ashley, Yoel Lubell

**Affiliations:** 1 London School of Hygiene and Tropical Medicine, London, UK; 2 School of Tropical Medicine and Global Health, Nagasaki University, Nagasaki, Japan; 3 Faculty of Public Health, Mahidol University, Bangkok, Thailand; 4 Lao Tropical and Public Health Institute, Ministry of Health, Vientiane, Lao People’s Democratic Republic; 5 Lao-Oxford-Mahosot Hospital-Wellcome Trust Research Unit (LOMWRU), Vientiane, Lao People’s Democratic Republic; 6 Mahidol-Oxford Tropical Medicine Research Unit (MORU), Bangkok, Thailand; 7 Centre for Tropical Medicine and Global Health, University of Oxford, Oxford, UK; 8 Faculty of Medicine, University of Queensland, Brisbane, Australia; 9 Oxford Medical Research Unit, Angkor Hospital for Children, Siem Reap, Cambodia; 10 Public Health Office, Savannakhet, Lao People’s Democratic Republic; 11 Institute of Research and Education Development, University of Health Sciences, Ministry of Health, Vientiane, Lao People’s Democratic Republic

**Keywords:** Laos, Community health workers, Primary care, Ethnic minorities

## Abstract

**Background:**

Community-based health programmes have been a cornerstone of primary care in Laos for decades. The study presented here aimed to document prospects for the development of current programmes, considering perceptions about health and health care priorities in the communities, implementation challenges, the policy landscape and opportunities associated with the availability of new technologies.

**Methods:**

The research design primarily involved qualitative in-depth interviews with stakeholders (n = 35) responsible for the planning, management, or implementation of community-based care in Laos at different levels of the health system. These included health managers at central departments or institutes of the Ministry of Health, provincial health departments, district health offices, heads of health centres, village health volunteers, community representatives, and international stakeholders.

**Results:**

There was consensus that service delivery is still a challenge in many areas, due to geographic inaccessibility of health facilities, communication barriers, health-seeking behaviour, trust, and gender discrimination, particularly among ethnic minorities. In these settings, community health workers have the potential to extend the reach of the formal health system, acting as cultural brokers across sectors of society, ethnicities, and worldviews. To maximise impact, planners need to carefully consider the implementation model, financing arrangements, health system integration, and changing health priorities in the communities.

**Conclusions:**

This study examined challenges to, and opportunities for, the expansion and health system integration of community-based care in Laos. Further development and horizontal integration of community-based care remains a complex financing and governance challenge, although the renewed emphasis on primary care and the ongoing process of decentralisation provide a favourable policy environment in the country to sustain and potentially expand existing programmes.

**Supplementary Information:**

The online version contains supplementary material available at 10.1186/s12913-023-10523-6.

## Introduction

Despite global progress towards universal health coverage (UHC), communities worldwide are still underserved by public health services [1]. In many countries, this constraint is most apparent in remote rural areas, where access to health information and care is difficult and where poorer communities bear the greatest health and economic burden of disease [2]. In these settings, people tend to self-medicate, forego medical consultation, seek care too late, or resort to inappropriate strategies such as using antibiotics for minor ailments or viral infections - a practice that contributes to antimicrobial resistance [3].

One approach to address these challenges is the involvement of lay health workers, variously known as “community health workers” (CHWs), “village health workers” (VHWs), or “village health volunteers” (VHVs), to provide health information and services in their communities. In Thailand, for example, the Ministry of Public Health manages more than one million village health volunteers across the country, providing them with a monthly stipend (US$32) to support local health centres in health education activities, disease surveillance, and referrals of patients [4]. Large community-based programmes have been established in other countries including Bangladesh, Brazil, Iran, Ethiopia, Nepal, and Pakistan [5].

In many low- and middle-income countries (LMICs), community-based care also receives substantial support from international organisations and donors, who generally favour a vertical approach focused on specific health issues in line with the global health agenda [6]. In Southeast Asia, the Global Fund has disbursed large grants to sustain “village malaria workers” (VMWs), trained to distribute bed nets, diagnose suspected malaria cases in endemic areas by using a rapid diagnostic test (RDT), administer artemisinin-based combination therapy (ACT), and refer patients with severe malaria to public health facilities [7–9]. Since 2018, these programmes have been a key component of the RAI3-Elimination programme (RAI3E), a US$ 228 million regional grant established to accelerate the elimination of *P. falciparum* malaria in the Greater Mekong Subregion [10]. Other issues that have been the focus of donor-funded interventions include maternal health, malnutrition, HIV, and tuberculosis (TB) [11].

While positive impacts of these programmes are well documented [12, 13], uncertainties and questions about their future have emerged [14]. In many contexts, health needs and priorities are changing, requiring adaptation of vertical programmes to ensure their continued relevance and uptake. In Southeast Asia, for example, malaria transmission has reduced considerably over the past decade and most acutely febrile patients will test negative for malaria, even in endemic areas [15]. However, VMWs have little guidance or capacity for effective management of non-malarial illnesses. In communities where malaria is no longer perceived to be a common cause of illness, utilization of VMWs may decline, especially if they are unable to care for the vast majority of patients with a negative RDT [16]. In addition, gaps in resources remain a significant constraint to the implementation of national and vertical programmes, exacerbated by the adverse economic effects of the COVID-19 pandemic on global health financing [17]. This is particularly true in countries where programmes are dependent on external support and resources. In these countries, therefore, a key policy question is whether these programmes should only exist as emergency interventions, or should they rather be sustained and potentially expanded to provide a more comprehensive package of primary services, also considering the availability of new, low-cost technologies for community-based testing and health monitoring [18–20]. If an upgrade of the current model is recommended, health planners need to consider the underlying governance arrangements and whether the new roles and tasks are seen as “acceptable and appropriate by their target population, the CHWs themselves, and those who support them” [21].

Considering these questions, we present here findings from a qualitative study which aimed to examine prospects for the development of community-based care in Laos. To this end, we explored stakeholders’ views and experiences about changing public health needs in remote areas, the recent implementation of community-based programmes and their views about the future of these programmes. Improving equitable access to health services in Laos has been a challenge for many years due to limited health care resources and its geography, characterised by forested mountains and steep terrain in large parts of the country [22]. While rural to urban migration has increased [23], Laos is one of the least urbanised countries in Asia and many people live in remote areas with poor road infrastructure. As of 2022, it was estimated that 62% of its total population of 7.5 million lived in rural areas [24]. In remote locations, travel from the villages to the nearest health centre is often difficult and time consuming, particularly during the rainy season. In addition, many remote villages are populated by ethnic minorities that face cultural and linguistic barriers to the use of public health facilities and other social services [25]. To reach these populations, community-based health programs have been implemented in Laos since the 1980s using different approaches and strategies. Today, the Ministry of Health is responsible for the national framework on primary care – involving health facilities and a large network of VHVs, available in almost every village throughout the country. The national framework coexists with a variety of programmes funded by different donors, including VMWs supported by the Global Fund [9, 26]. Recently, interest in community-based care has increased after the revision of the Policy on Primary Care (2000), issued in 2021, in recognition of persisting poor health outcomes in remote areas. However, questions remain about the best governance and implementation arrangements to support community-based care in the country as well as the role and integration of donor-funded vertical programmes within the national framework. These questions are also due to the health care implications of the epidemiological transition. Back in the 1990s, nearly 69% of disease burden in Laos was associated with communicable diseases, while non-communicable diseases (NCDs) contributed only 24%. Over time, as in other LMICs, the prevalence of infectious diseases has decreased steadily. By 2016, NCDs were estimated to account for 47% of disease burden, surpassing the 43% attributed to communicable diseases [27]. In this context of change, we examined challenges to, and opportunities for, the expansion and sustainability of community-based care in Laos through interviews with a wide range of domestic and international stakeholders.

## Methods

### Research design

This study is part of a large multi-disciplinary research project - the South and Southeast Asian Community-based Trials Network (SEACTN) - which aims to assess and test interventions to address the burden of disease among disadvantaged population groups in rural areas of Bangladesh, Cambodia, Myanmar, Thailand, and Laos [28]. Here, we present and analyse findings from qualitative interviews with stakeholders involved in planning or implementing community health programmes in Laos. Following Brugha and Varvasovszky [29], we defined stakeholders as “actors who have an interest in the issue under consideration, who are affected by the issue, or who – because of their position – have or could have an active or passive influence on the decision-making and implementation processes”. In the research design, we recognised that any plans for the development of community-based care needs to consider carefully the evolving public health needs as well as lessons learned from recent experiences. Consequently, the interviews aimed to cover the following topic areas: (1) public health priorities in remote areas; (2) challenges to health service delivery in these locations; and (3) the current role and prospects of community-based care (Additional File 1). We also acknowledged that improving access to care in remote communities is a complex policy issue, affecting and being affected by different actors with their own ideas, interests, needs, experiences, and influence on decision-making and implementation processes [30].

To account for this diversity, we aimed to recruit a wide range of participants across the public health sector, local communities, and international organisations. In the public health sector, following preliminary consultations with experts to map key stakeholders, interviews were conducted with health professionals responsible for the planning, management, or implementation of community care at different levels – central departments or institutes of the Ministry of Health, provincial health departments, district health offices, and health centres. In keeping with bottom-up approaches to policy analysis [31], we also aimed to achieve a fair representation of the local communities and gender, including village authorities, representatives of the Lao Women’s Union, and VHVs. Villages were purposively selected in remote areas of two provinces in the southern part of the country, with endemic malaria and inhabited by Lao, Mungkong, and Kathang ethnic groups. In addition to domestic stakeholders, community-based care in Laos involves many international organisations, consultants, and non-governmental organisations (NGOs). Thus, interviews were also conducted with representatives of these organisations and researchers with extensive knowledge of the health sector in Laos, identified through the professional network of the authors and snowball sampling.

Given the diversity of the study sample, the interview schedule was adapted to the role and expertise of different stakeholders and refined in an iterative process following pilot interviews across all categories of stakeholders. Questions related to the present and future of community care in Laos were informed by recent contributions in health system research that identified key areas for systematic investigation, including roles and tasks, governance, financing, incentives, training, relationships with the health system, and the communities [14, 30, 32]. Considering the potential of new technologies for community-based care, specific questions were included about the use and impact of RDTs and whether new diagnostics or health monitoring tools should be introduced to expand the scope of current programmes.

### Data collection

Following interviews with an initial set of stakeholders, additional participants were recruited by snowball sampling or purposively selected to explore emerging issues further. In-depth interviews with international stakeholders were conducted by the first author in English, while the other interviews were conducted in Lao by KP and three researchers from the Lao Tropical & Public Health Institute, all with training and previous experience in qualitative research methods. Part of the interviews with central-level and international stakeholders were conducted online due to COVID-19 restrictions, while the other interviews were conducted in person. Interviews lasted between 30 and 90 min and, if consent was given, were audio-recorded. Transcripts of interviews in Lao were translated into English and verified for accuracy of translation through iterations with the researchers who conducted the interviews. In the villages, unstructured field notes were also taken to record observations of social and health practices. Information on the aims and objectives of the research project was provided to all participants and consent was obtained prior to being interviewed. All data were anonymised and stored in a secure archive, in compliance with the research protocol approved by the National Ethics Committee for Health Research of Lao PDR and the Oxford Tropical Research Ethics Committee. Throughout the project, policy documents, previous studies and reports were searched and reviewed to provide contextual information and clarify emerging points/themes from the interviews.

### Data analysis

Raw data from the interviews and the interview notes were organised and processed in a systematic way to identify and explore themes, issues, and relationships. Drawing on established methods in qualitative research and policy analysis [33, 34], an analytical framework was initially developed using the domains and categories described earlier. The framework was then used for selective coding of the dataset, aided by the software NVivo 13 (QSR International). The analysis also involved open coding to enable a wider reading of the dataset, identify emerging themes, and refine the categories in the analytical framework. Subsequently, we generated analytical summaries for each category to represent wider ideas and concepts within the data, convergence of themes/concerns, potential tensions, and conflicts [35]. In the summaries, references to the sources and illustrative quotations were retained and used in the presentation of findings below.

## Results

### Overview

Between June 2021 and July 2022, we conducted interviews with 35 respondents; 28 were individual interviews and two were group discussions involving seven participants. As summarised in Table 1, participants included managers in central departments of the Ministry of Health and associated institutes (n = 4), international stakeholders (n = 4), directors/officers at the provincial and district level (n = 6), heads of primary health centres (n = 2), VHVs (n = 4), and village authorities including heads/deputy heads of village (n = 6), and representatives of the Lao Women’s Union (n = 2). Interview meetings were attended by individual participants (n = 28) or a small group of (3 to 6) participants working in the same organisation. None refused to participate in the study or dropped out during the interview. In the presentation of findings below, structured around the four key dimensions in our investigation, emerging themes and anonymised citations are referenced by the unique identifiers described in Table 1.

**Table 1:** Summary of interview participants, with unique identifiers

**Central level MOH**	Directors/deputy directors of MOH centres or institutes (CL01, CL02, CL03, CL05)
**Provincial/district level**	Directors/deputy directors of provincial health departments (SP01, CP01); health managers at district health offices (SP02, SP03, CP02, CP03)
**Communities**	Village head / deputy head (SP08, SP09, CP06, CP08, CP09, CP10), VHVs/VHWs (SP05, SP06, CP05, CP07), representatives of the Lao Women’s Union (SP07, CP04), head of health centre (SP04, SP10)
**International stakeholders**	Representatives of international organisations (II01, II02, II03), researchers (II04)
**Group interviews**	Domestic stakeholders (G01), international stakeholders (G02)

### Views and experiences about health needs in the communities

One of the objectives of our study was to explore perspectives across the health sector on priority public health needs in remote communities in Laos. During the interviews, domestic and international stakeholders mentioned different conditions and concerns, bringing into sharp focus the challenges of providing health care to ethnic minorities. One international expert noted that “the farther you get from the capital, the worse the outcomes” and went on to explain:

Many ethnic groups are not doing so well compared to the ethnic majority. You see those populations that are remote without roads are also not doing so well on several indicators, whether it is health outcomes or access to services (II03)

Specific challenges related to maternal and child health were discussed in many interviews. In both provinces, 20 to 30% of deliveries were reported to occur at home due to cultural preferences (SP09, CP10, CP02) and deliveries on the road to the health centres were reported in three out of six villages in our sample. Aware of these issues, local stakeholders and international partners are working together to promote antenatal care and safe delivery and improve nutrition (CP02, II02). Yet there were strong views that more investments are needed to save the lives of mothers and children. The country director of an international organisation stressed that “maternal and child health is probably the priority number one; until mothers stop dying in childbirth, until numbers decrease” and explained:

When you look at the Lao health system, the first thing to say is that progress in maternal mortality and child mortality under five remains far below other countries with similar GDP per capita, driven largely by inequities between the wealthiest and the poor…. exacerbated in the huge number of ethnic minorities (II02)

Febrile illness and diarrhoeal disease were also concerns in the communities as village authorities and VHVs often mentioned episodes of “fever”, “fever with chills”, and “stomach problems”. However, malaria – a major public health issue in Laos for many years - was no longer seen as a top priority. In two villages near the forest, sporadic malaria cases were reported and in other villages they had not seen malaria for months (CP10) or even years (CP05) In one of them, still classified as endemic, the VHV had forgotten how RDTs work after years of non-use.

These views and experiences at the community level were mirrored in the accounts of stakeholders, who recognised the importance of malaria elimination but also the need to shift attention and resources to other infectious diseases such as TB, dengue, and to non-communicable disease (NCDs) and associated risk factors, particularly “diabetes and hypertension” (II02):

Many people look down on TB… they think it is not important enough, not really severe. But I want other researchers to pay attention to tuberculosis as well... if we compare the mortality rates of malaria and tuberculosis [in Laos], we can see that tuberculosis continues to increase year by year (CL04)

We need to focus on non-malarial diseases, particularly dengue fever… This is a big concern. In the villages, they even forget malaria… they say malaria is not important, not an urgent need like dengue fever… they are also concerned with TB, diarrhoea, pneumonia… they occur a lot in the communities (GD01)

Other infectious diseases that were cited in our interviews included Chikungunya, leptospirosis, and rickettsioses (II04, G02).

### Access to health facilities

#### Socio-cultural factors

When discussing challenges in access to care, many participants mentioned cultural practices and beliefs of ethnic minorities and their influence on care seeking behaviour. Laos is ethnically diverse, with 49 ethnic groups recognised by the government [36]. Many of these groups speak their own language, which may not be mutually intelligible with Lao, the official national language. One example is Hmong, spoken by approximately half a million people in the country. Due to these language barriers, communications between health workers and patients from ethnic minorities are often challenging, particularly for the elderly, women, and those who did not attend formal school education (SP04, SP04b, SP10, SP03a, CL03, II04, CP03). As the deputy director of a provincial health department pointed out, these constraints are not only an issue when health workers need to interact with the patients regarding diagnosis and treatment, but they may also discourage service utilisation and undermine trust in the public health sector (SP01c). Similarly, an international expert commented:

So what you find is there is a lack of trust in the health centres because they don’t speak their language… even if the health centre is near their village, they may not immunise their children or women may not deliver in those facilities because they don’t trust them… so it’s not all about building roads or building health facilities closer to the population; it’s also about seeing how we can address these factors and link these populations to the health centres (II03)

In these communities, health seeking behaviour is also influenced by the widespread belief in the healing powers of spirits and rituals (SP06 VHV, SP09, CL02 MOH, CL03 MOH, CL05, SP01b). During one of our visits, the local healer played a pipe instrument and recited prayers to heal a young woman with fever and severe headache. In another location, the village chief explained that pregnant women like to deliver in the comfort of their home, where their mothers cut the umbilical cord as part of a ritual to protect the wellbeing of the child (SP03a). In the interviews, local stakeholders acknowledged these customs respond to important social and psychological functions in the communities, but they were also concerned about their health implications if people engage in hazardous practices and forego or delay medical care. In some communities these issues are compounded by social norms and gender discrimination, preventing women to visit the health centre if their husbands disagree (SP04, CL05).

#### Geographic accessibility

Since the early 1990s, the government of Lao PDR has committed to providing affordable health care and education for the whole population. As a result, the health sector is large, relative to a population of 7.5 million, with 5 central hospitals, 17 provincial hospitals, 135 district hospitals and 1074 health centres. Since 2016, another major step in the path towards universal health coverage in Laos has been the introduction of the National Health Insurance (NHI), which aims to cover all Lao citizens outside Vientiane capital, including those working in the informal sector and the poor. Members can access public health services by contributing small co-payments - LAK 5,000 (US$ 0.3) for outpatient care at health centres and LAK 15,000 (US$ 0.7) for inpatient care at district hospitals. Health care is free for pregnant women, children, and the poor, defined as those who earn less than LAK 280,910 (US$ 16.2) per month (LSB 2020). Following the introduction of the NHI, social health protection has increased considerably in the country - from 10.5% in 2008 to over 94% of the total population in 2018, according to official data [37].

Despite much progress, there was a consensus among stakeholders that health care delivery remains a challenge in remote areas (II01). While geographic accessibility has improved throughout the country (CP09, SP02b), traveling to the nearest health centre is still difficult from many locations due to the rugged terrain and poor road infrastructure, as we experienced in our field visits. The deputy director of a provincial health department estimated that 3% of villages in the province are still isolated from health facilities and other social services (SP01c). In these locations, the travel costs to reach the health facilities further discourage service utilisation (SP02, SP06).

#### Community-based programmes

In order to address these challenges, the government of Lao PDR and international partners have implemented community-based programmes involving lay health workers. VHVs were established in the early 1990s, when lay people were selected and trained to provide basic care in their communities, manage revolving drug funds (in which a small fee is charged for essential medicines and then used to purchase new medicines), and support the health centres in health promotion activities (II01). Following the initial implementation phase, VHVs have been expanded as part of the health and sanitation activities included in the “Model Healthy Villages” programme, financed by the Asian Development Bank and the Japan Fund for Poverty Reduction [38]. Over time, VHVs have been increasingly institutionalized, becoming part of the national public health infrastructure (Sato et al. 2014) [39]. In 2005, the Law on Health Care (2005) remarked at the highest policy level the importance of community-based care and village drug kits, managed by “village assistant physicians or village public-health volunteers and traditional birth attendants” that “provide drugs and offer consultations and treatment for benign seasonal illnesses, in particular diarrhoea, malaria, flu, and minor wounds, assist in home births, and distribute medicine” (Article 14). One international expert explained that the public health value of VHVs has been further recognised after the WHO-UNICEF global conference on primary care in Astana, Kazakhstan, where the Lao delegation signed with other countries a declaration of commitments, including building “sustainable primary health care” [40]. At that time, there was a strong awareness that vulnerable groups were suffering a disproportionate disease burden in Laos, so “they wanted to focus more on community-based primary health care” (II03).

**Fig. 1:**
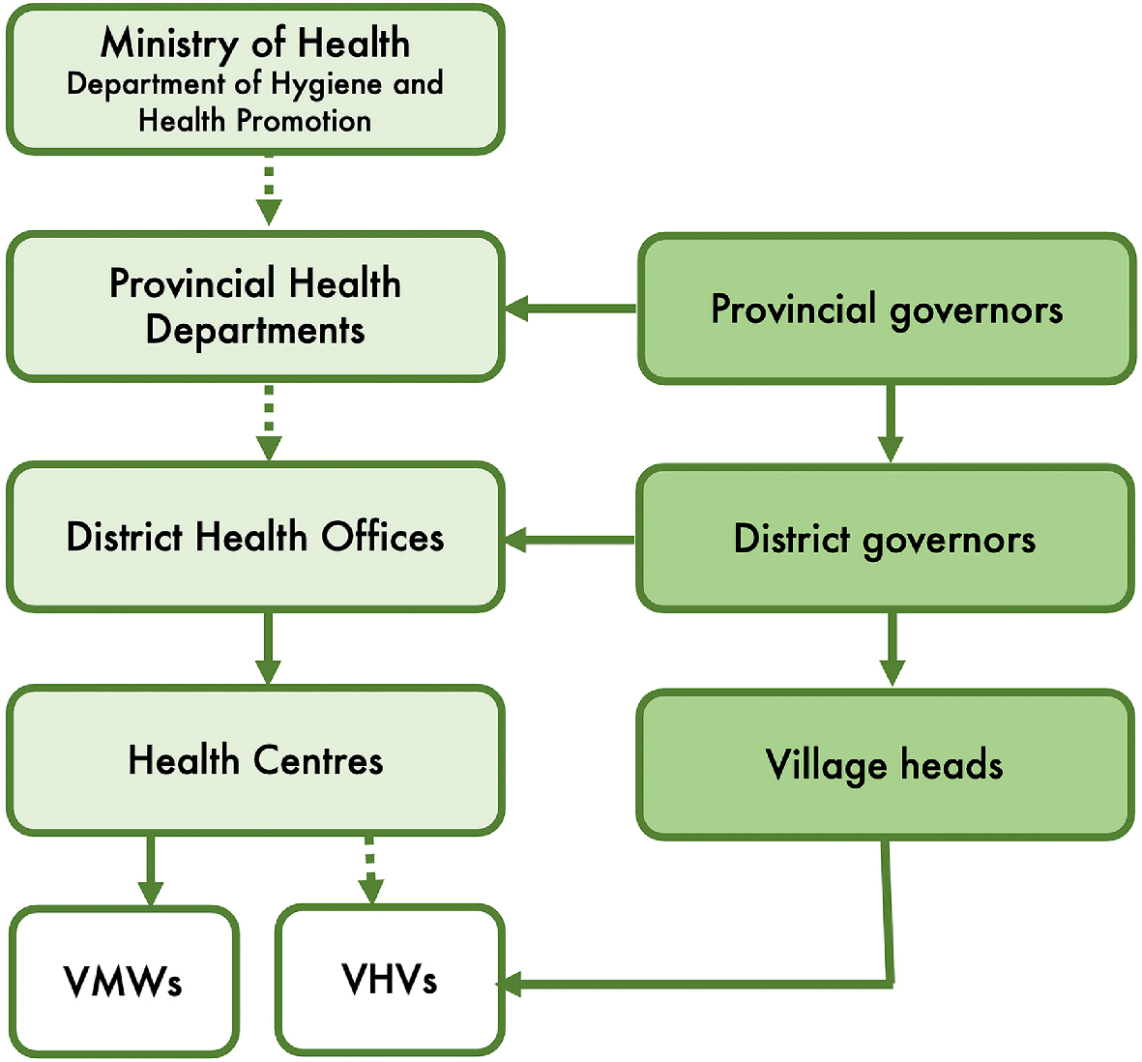
Organisation of community-based health services (VHVs and VMWs) in Lao PDR. Solid lines indicate hierarchical relations. Dotted lines indicate responsibility for supervision and monitoring

Today, VHVs are a key component in the national Health Sector Reform Strategy and Framework 2016–2025 (MOH 2016) and the Policy on Primary Care (2021), which was recently revised and maintained a key public health role for VHVs. At the central level, the programme is managed by the Department of Hygiene and Health Promotion, in collaboration with Provincial Health Departments, District Health Offices, and the health centres. At the community level, VHVs are embedded in the local governance structure – the village committee - which also includes the village head, the deputy head, the head of security, a representative of the Lao Women’s Union, and a Youth representative (Fig. 1). VHVs are typically selected and appointed by the village head in consultation with the local district authority; thus, as participants noted, the decision is “made outside the health sector” and not by village election (II03, SP05). In fact, in many villages, VHVs are the authorities themselves (including head and deputy heads) or their kins, who often fulfil this role for many years along with their main administrative duties (SP01c). Following appointment, VHVs receive basic training and are expected to support their health centre in various activities including preventive care, health promotion, treatment of common and widespread illnesses, and referral of patients.

In addition to VHVs, international organisations and donors have supported vertical programmes in the communities, focused on particular health issues such as malaria, maternal and child health, nutrition, and water management. The malaria programme, involving the deployment of health volunteers with specialised malaria training (referred to henceforth as Village Malaria Workers or VMWs), was established by the Global Fund in 2008 as part of a wider package of interventions to reduce malaria. As other donor-funded programmes, VMWs are different to VHVs in terms of resources, governance, and implementation model. In some provinces, local VHVs are also appointed to the role of VMWs, but in many others VHVs and VMWs are different persons, selected using different criteria. While hierarchies and status play an important part in the appointment of VHVs, VMWs are recruited by the implementing organisations in consultation with health authorities, with a view to achieving gender balance and representation of local communities. Importantly, VHVs are indeed *volunteers*, receiving no regular compensation for their work; by contrast, VMWs receive a monthly stipend of about US$13 for their contribution to vector control activities, disease surveillance, case management using RDTs and ACT, and referral of patients with severe malaria to the health centres they are linked to [22]. In 2020, there were 13,722 VHVs [41] and 2,576 VMWs [42]. VMWs are deployed only in areas classified as high malaria transmission in the southern part of the country.

#### Implementation

In the study settings, implementation of community-based programmes was variable, depending on local resources, approach, and type of programme. In some locations, VHVs were involved in multiple activities such as treatment of colds and other minor illnesses; reporting of births, deaths, and outbreaks; sanitation and water management; health promotion; management of the revolving drug fund; support to the COVID-19 response through monitoring quarantine, distribution of supplies, and surveillance; vaccination programmes, sanitation; and malaria testing and treatment for those involved in the malaria programme. In one village, the local VHV had expanded his role well beyond the terms of reference, building a reputation for treating sciatica and attracting forest workers with back pain from neighbouring villages (CP07). In another village in the same province, we observed the local VHV, a former paramedic, injecting intravenous fluids to a woman in pain. The head of village explained that “some people who have diarrhoea want the VHV to give them infusion, so he takes the infusion from the health centre and bring it here” (CP06b). It was also mentioned that VHVs may sell antibiotics, despite this is not allowed by law (SP04). In other locations, however, VHVs were not really active, and uptake was reportedly low. In these settings, concerns emerged about late or no reporting to the health centres, lack of motivation, and lack of trust by the communities in the ability of VHVs to provide reliable advice and care.

When discussing factors explaining these outcomes, there was a perception that in many villages VHVs lack motivation and their assignment has low priority in the daily schedule as they need to attend to other things for a living (SP02, SP06, II01). In other villages, as described, VHVs function as (unauthorised) private providers, charging a small fee to offer additional services and thus compensate for lack of regular financial incentives. VHVs also tend to be males as men are those undertaking public roles in these groups (SP09), while young women VHVs may be forced to drop their assignment after marriage due to the gender discriminations described earlier – a major limitation when it comes to maternal health services. As the deputy director in a provincial health department said: “here women don’t want to talk to a man about menstruation and pregnancy” (SP01b).

Implementation challenges of vertical programmes also emerged. While the monthly stipend for VMWs provides a performance incentive, according to many stakeholders it is insufficient to curb high attrition rates among VMWs as they look for other opportunities to make ends meet (SP01b, SP02 DHO). Furthermore, it was noted that the allocation of stipend to VMWs has the unintended consequence of frustrating the motivation of VHVs, who often complain about unfair treatment (SP04b, SP01a):

Malaria workers receive a monthly salary, unlike VHVs… If VHVs and VMWs live in the same area, they may know each other… and this create tension between them. VHVs resent they don’t receive any payments. We should also support VHVs because they are both working for the people… this is a big challenge (SP01a)

### Prospects

In our conversations about the future of these programmes, there was wide agreement across all categories of stakeholders that community health workers remain an important asset in Laos and should be sustained to reduce the inequities in access to services described earlier, particularly in remote areas and among ethnic minorities. In both provinces, it was also noted that the number of VHVs should be increased to cover more efficiently their catchment areas (SP06, CP09, SP06), while the appointment of VHVs should be revised in line with the VMW approach to make sure they are representative of their communities, speak Lao as well as the language of their ethnic group, and there is a larger representation of female volunteers (CP02, II03). There was also agreement that the reward system should be improved (II02).

#### Expanded services and technology adoption

Stakeholders were positive about the potential expansion of vertical programmes (CP06b, SP06), suggesting this would make them more relevant to the current public health needs in their communities (SP04, SP01a). Specific add-on services that were proposed include rapid testing for COVID-19, dengue, rickettsial disease, and leptospirosis (SP01a; SP02); smartphone apps to provide advice on maternal health and nutrition (CP02b, G02); and blood pressure and glucose monitoring for the prevention and control of NCDs (II02). Furthermore, both central-level and international stakeholders emphasised the importance of adaptation to different contexts, with a focus on health system integration and infrastructure:

It would be good if we use RDT tests for other diseases, especially in remote areas… and the outreach teams can also use them instead of carrying many things to do the diagnoses. But we don’t know how we can manage these findings… For example, we have the COVID-19 test: after we know the test results, what do we do? The same is for the glucose test… We need to clarify the role of the health centres and the district level (CL01)

So the question for us is how we differentiate and operationalise [expansion of services] in a meaningful way… from my perspective how the village and each village gets the right set of activities or diagnostic whatever for the context of that village in a way that is quality, well-supervised and linked to the health center in an effective way…and I don’t think I have any idea on how to answer that question yet… (II02)

Most VHVs have a normal mobile phone, but no smartphone… signal is only available in certain areas, maybe outside the village… they walk there, they switch the phone on and then off again when they are in the village (SP04)

It was also mentioned that plans for expanding the package of services need to account for the limitations of lay health workers in terms of literacy and familiarity with technologies, requiring easy-of-use devices/tools and approachable training material, especially for the older generation (SP10, CP03, CL01, G01, G02, II04). In a group discussion participants suggested that expansion of testing services should be limited to two or three priority diseases such as “malaria, dengue, and COVID-19” (G02). Based on past experiences, another participant recommended avoiding heavy loads of written text and using instead visual training materials (II02).

#### The policy landscape

An assessment of prospects for the future of community-based care in Laos should also consider the wider policy landscape, including the national governance framework and the perspective of international actors. In 2021, the Lao government approved the revised Policy on Primary Care, which emphasised the importance of community-based care, and provided a favourable environment for the continued development of health volunteers (G01). As one international expert pointed out, this approach is in line with the process of decentralisation and the “3-Build” (*sam sang*) policy, a bottom-up governance framework which aims to improve public services in the communities through delegation of responsibilities and the empowerment of local administrations [43]: “the Ministry of Health has a very clear set of policies around strengthening primary health care and the new five-year plan was finalised just earlier this year… this places a strong emphasis on district hospital, health centres, and community level care” (II02). It must also be noted that the Ministry of Health recently endorsed the Digital Health Strategy 2023–2027, which emphasises “the untapped potential for both village chiefs and village health volunteers to play a bigger role in Lao PDR’s digital health ecosystem” [44]. At the same time, there was a perception that the MOH envisages VHVs primarily as a link between the communities and the formal health system, rather than undertaking the role of formal health workers or some of their medical duties (II01, II02, II04).

In parallel to these reflections, it was stressed that financing issues should be carefully considered. The potential upgrade of VHVs to the status of paid civil servants would require substantial commitment from the government, including the payment of monthly stipends for thousands of VHVs. Based on past experiences, stakeholders believed this is unlikely to happen in the short term. In 2014, for example, VHVs in pilot villages were trained for six months to become paid “Village Health Workers” with a wider range of skills and responsibilities such as maternal and child health, treatment of common colds, pneumonia (with antibiotics), malaria, diarrhoea, deworming, skin infections, and family planning services. The pilot project received support from ADB, but lack of domestic financing prevented scaling up and health system integration. Today, the situation is even more difficult due to the adverse economic consequences of the COVID-19 pandemic, resulting in cuts to an already strained health budget (CL01). Furthermore, two international stakeholders explained that the Lao government was pressured by global financing organisations – particularly the World Bank and the International Monetary Fund – to reduce the number of civil servants (II01, II03). As a result, “every year the MOH only have an additional 300 or 400 staff for the whole health sector… which means that the government cannot easily have 14,000 new staff on its payroll; this would double the number of current MOH employees” (II01).

In the discussions about these prospects, two scenarios emerged as potential ways forward to support the development of community health care in Laos. The first scenario focuses on domestic funding at the local level, leveraging opportunities associated with the ongoing process of administrative decentralisation. While the central health budget is “seriously diluted by the time it comes to the village level” (II02), the provinces and the village leadership may be able to sustain health volunteers if their value is recognised by the communities. In fact, at the time of this study, plans were being discussed in both provinces to support VHVs through the provincial budgets (CP02, SP02b), with monthly compensation in the range of LKP 200–300,000 (US$ 12–17). In one province, the deputy director suggested that additional rewards could be provided in the form of free care without co-payment and recognition of performance through awards (SP01).

The second scenario entails sustained investments by donors. International stakeholders agreed that major global health actors recognise the need for more investments in primary care and new health priorities, as illustrated by the emergency funds for COVID-19 prevention and control [45]. It was also mentioned that primary care has received more attention and resources recently, in the context of initiatives towards the achievement of the Sustainable Development Goals and Universal Health Coverage. At the same time, stakeholders were aware of constraints in donor financing and its precarious nature, at a time of economic downturn. There was also recognition that scale up and wide adoption of upgraded programmes would require a better understanding of challenges to health system integration and more evidence about the impact and uptake of new technologies. The representative of an international organisation explained:

RAI [i.e. the Global Fund] is a conservative institution… they are not willing to add new diagnostic tools until we demonstrate they are meaningful and useful so we are hoping to get some findings to show that… and then say this will now be part of the VHV package… not just malaria test… so we should have an algorithm… should malaria be negative (which is 99% of the times), what do they do next? (II02)

## Discussion

This study explored prospects for the development of community-based care in Laos, focusing on public health needs in remote communities, implementation strategies, and the underlying governance and financing arrangements. As described, there was a consensus among domestic and international stakeholders that service delivery is still difficult in many areas, due to geographic inaccessibility of health facilities and associated costs, communication barriers and health-seeking behaviour, trust, and gender issues. In these settings, CHWs have great potential to extend the reach of the formal health system, acting as cultural brokers across sectors of society, ethnicities, and worldviews. However, our study also highlighted that programme design and implementation are complex tasks, requiring a consideration of constraints and opportunities at different health system levels (Table 2).

**Table 2:** Prospects and recommendations for the development of community-based care in Laos

**Expanded roles and tasks**	Potential expansion of testing services for non-malarial febrile illness and NCDs prevention, using low-cost, user-friendly diagnostic tools; smartphone applications and guidelines for antenatal care and the management of childhood illness
**Training**	Focus on audio-visual educational programmes; digital literacy
**Incentives**	Monthly stipend; access to health care without co-payment; awards
**Relationship with the communities**	Focus on selection criteria, considering gender balance, bilingual skills, and education
**Relationship with the health system**	Programme adaptation to different contexts and needs; horizontal integration of vertical programmes; technology adoption
**Financing**	Continued donor support, while planning for local funding through provincial budgets and village funds
**Governance**	Increasing devolution of responsibilities for planning and management to provincial health departments and district health offices

Based on the findings, a first consideration is that the national framework is a well-established institutional platform to organise community-based care in Laos, but financing challenges remain. As a result, there is a lack of resources to support key programme components such as financial incentives for VHVs, continued training, and reporting to health facilities. As documented in other contexts [46], lack of financial incentives for community health workers is a major constraint to effective programme implementation, which in our case also resulted in tensions with donor-funded programmes. Another limitation of the national programme is that the appointment of VHVs tends to be determined by social status and gender hierarchies more than effective selection criteria. In particular, female VHVs represent only 40% of volunteers. As seen in other countries, programme uptake and impact can be increased through the involvement of more women in health programmes [47, 48]. In Bangladesh, for example, the national programme managed by BRAC has involved a large number of female community health workers (*shasthya shebika*), leading to substantial improvements in maternal health and other indicators, while empowering women as agents of change in their communities [49]. In Laos, a more balanced gender representation could be achieved nationwide through the involvement of the representatives of the Lao Women’s Union to take on the role of VHVs, as already occurring in some communities.

A second consideration is that, compared to the national framework, vertical programmes tend to have more resources and therefore can rely on a more effective implementation model. However, long-standing vertical programmes need adaptation and horizontal integration to respond to changing health priorities in the communities. As documented in neighbouring countries, specialised malaria workers are a critical public health asset in endemic areas to support malaria elimination, but they have lower value in villages where malaria incidence is low [8, 16]. By contrast, an expansion of these programmes to cover key primary health concerns was found to improve uptake and health outcomes for both common illnesses and malaria [16].

At present, as we have seen, there are also good opportunities associated with the availability of new technologies for testing and health monitoring in the communities. In Laos, smartphone applications for antenatal care and the management of childhood illness would be particularly useful to address gaps in these key public health areas, as seen in other contexts [50, 51]. Portable testing tools for a variety of pathogens could also be used to inform case management of non-malarial febrile illness such as rickettsial disease and leptospirosis, combined with appropriate policies on antibiotic dispensing in the health centres and, potentially, in the communities [52, 53]. While stakeholders recognised the potential of such technologies, they also discussed various implementation challenges, particularly in relation to capacities and health system integration. As one health sector manager effectively summarised, “Once they have the results, what do they do next?”. This is a reminder that any plans for the introduction of new technologies should consider carefully human resources, infrastructure, and other factors that may affect adoption and sustained use [54]. In this respect, it must be noted that a report published in 2018 highlighted that access to mobile broadband is increasing, but rural and more remote communities are still unserved or underserved [55].

**Fig. 2:**
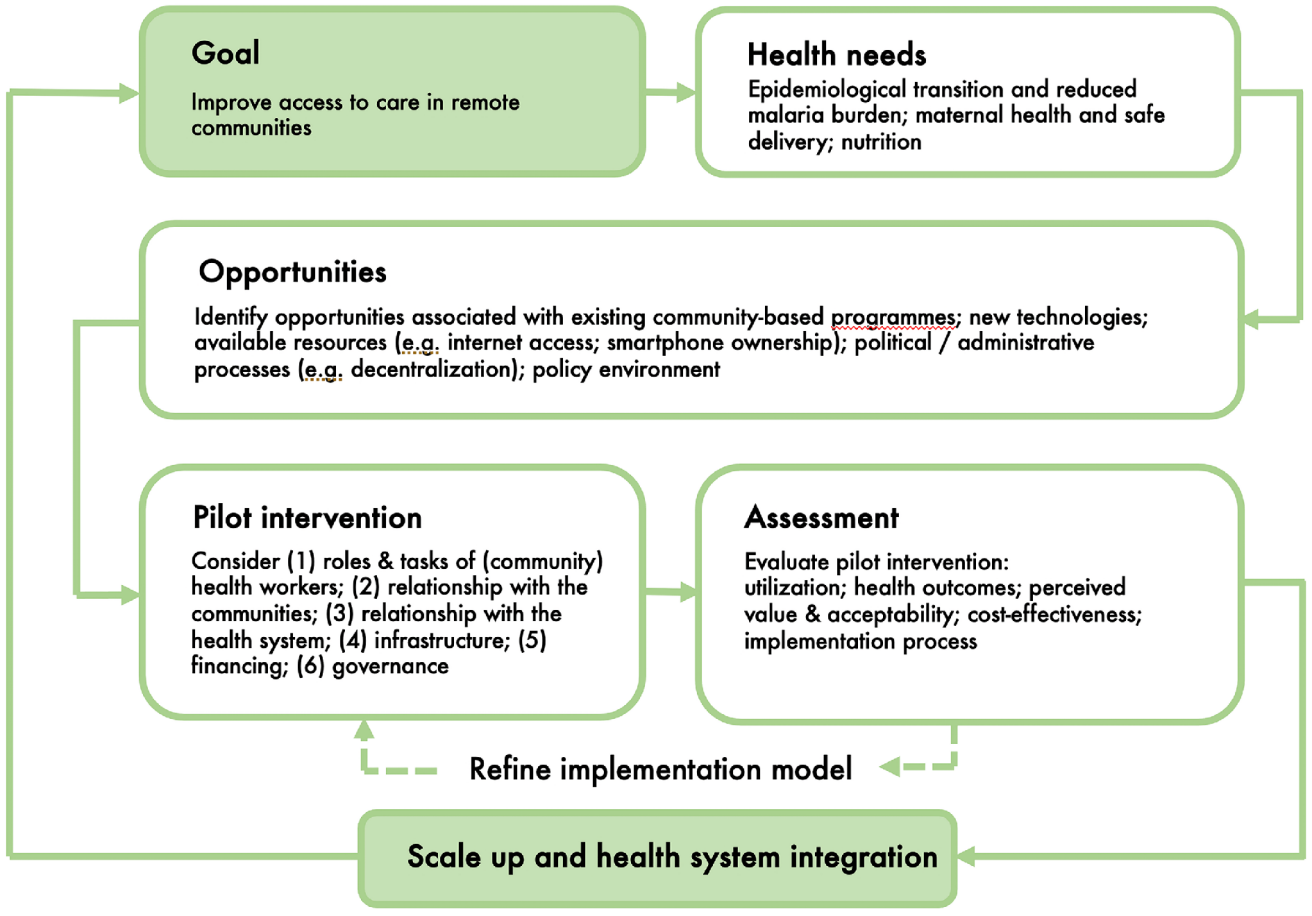
Visual representation of a strategy for developing community-based health programmes in Laos, from the definition of the agreed goal and priorities to the design, assessment, scale up, and health system integration of adapted/expanded interventions

Lastly, the analysis of the policy landscape suggests there is a convergence of favourable developments in support of community-based care in Laos. As we have seen, the current emphasis on primary care in national and global policy statements [40, 56, 57] opens a “window of opportunity” to sustain and expand existing community programmes [58]. In Laos, the process of decentralisation within the wider framework of the “3-Build” policy also enhances local ownership of social services, providing a more sustainable foundation for the development of community-based care. At the same time, further health system integration of VHVs into primary care faces major challenges such as the limitations to the quotas of civil servants, requiring legal and regulatory adjustments. Domestic funds to support and sustain the expansion of community-based programmes are also limited. The economy of Laos has faced unprecedented financial difficulties in recent years, including US$14.5 billion worth of foreign debt. Adding to this, the COVID-19 pandemic has hit the national economy hard, increasing concerns about the risk of debt default [59]. As a result, domestic funds to support the local health sector have shrunk although the national health insurance scheme has been rolled out, making health care more affordable and accessible in many provinces. Furthermore, the ongoing process of decentralisation provides unique opportunities to sustain and potentially expand community-based programmes in Laos. However, there are imbalances across regions, which may affect their ability to support these programmes. Recognising these challenges, international organisations and donors such as the ADB, the Global Fund, and national aid agencies are providing continued support to help economic and health sector recovery in Laos. In November 2020, the Global Fund announced a co-financing agreement with the World Bank and the Government of Australia to invest $36 million toward universal health coverage in Laos by 2025 [60]. Part of these funds could be allocated to design and implement pilot projects with expanded village health workers, capitalising on their in-depth knowledge of the communities and the availability of new, low-cost technologies. In turn, as illustrated in Fig. 2, evaluations of these project will provide more evidence about the uptake and impact of innovation in community-based care, which can be used to inform guidelines and potential scale up and integration, when the necessary resources and infrastructure will be available.

## Study limitations

This study examined perspectives and experiences of stakeholders around an important health policy issue, adding to recent efforts to better understand health care needs in remote communities in Laos [61, 62]. While the findings on health priorities in these communities provide in-depth insights and perspectives into local health challenges, they are not based on epidemiological studies. Thus, they should be read alongside, and triangulated with, evidence from other work that is currently being undertaken to provide an accurate mapping of the disease burden in these population groups through clinical studies, health surveys, and verbal autopsy methods [28]. We should also note that the study locations in southern Laos might not be representative of health priorities and challenges in other parts of the country. Furthermore, this study is the outcome of the analytical perspective of the authors, which may be skewed to emphasise particular issues and neglect others due to positionality biases [63]. As such, it is meant to inform the debate and future studies of community-based care in Laos and in other countries, rather than providing definite answers as to which services should be expanded in community settings. Finally, part of this study was conducted soon after the peak of the COVID-19 pandemic in Southeast Asia, with limitations to travel and the availability of stakeholders for the interviews; stakeholder views are likely to have been affected by the pandemic and the specific challenges health care services faced in their response to it.

## Conclusions

This study documents persisting challenges to health service delivery in rural Laos, associated with geographic inaccessibility of health facilities, cultural barriers, health-seeking behaviour, and gender discrimination, particularly among ethnic minorities. In these settings, community health workers have great potential to extend the reach of the formal health system, acting as brokers across sectors of society, ethnicities, and worldviews. Furthermore, there is a favourable policy environment to support the expansion of current programmes and the introduction of new technologies for community-based testing and case management. To maximise impact, planners need to carefully consider the implementation model, horizontal integration and changing health priorities in the communities. Further horizontal integration of community-based care remains a complex financing and governance task, although the renewed emphasis on primary care and the ongoing process of decentralisation provide a favourable policy environment to sustain and potentially expand existing programmes. Future research and evaluations of pilot projects will provide more evidence about the uptake and cost-effectiveness of expanded programmes, which can be used to inform guidelines and potential scale up in Laos and in other countries experiencing similar health system challenges.

### Electronic supplementary material

Below is the link to the electronic supplementary material.

**Additional file 1**: Interview guides. Translation of the qualitative interview guides.

## Data Availability

The full datasets generated and analysed during the current study are not publicly available due to confidentiality agreements but individual transcripts are available from the corresponding author on reasonable request for research purposes, and with permission of the interview participants.
