# Supplementary material for: Prospects for the development of community-based care in remote rural areas: a stakeholder analysis in Laos
Source: BMC Health Serv Res. 2024 Jan 11;24:55. doi: 10.1186/s12913-023-10523-6 (PMC10782664; doi:10.1186/s12913-023-10523-6)
Supplement: Supplementary file 1 — Additional file 1: Interview guides. Translation of the qualitative interview guides. [file 12913_2023_10523_MOESM1_ESM.docx]

**Additional file 1: Interview guides**

**1. Introduction and background**

Thank you very much for participating in this interview. Could you tell me something about your background and current work?

**2. Health needs in the communities**

This project focuses on health needs in remote rural communities in Laos. What are the most urgent priorities? Can you elaborate on this?

Probe:

- Encourage discussion of challenges associated with specific population groups (eg ethnic minorities, women)

**3. Health sector gaps**

As part of this project, we would like to gain a better understanding of challenges to health care delivery in these areas. What are your views?

Probe:

- Consider barriers in access to services across different access dimensions: geographic accessibility, availability, affordability, and acceptability

**4. Policy prospects**

What is the current policy framework to address these gaps? What are the plans for the future?

Probe:

- Discuss potential policy challenges but also opportunities (such as upcoming rounds of donor funding)

**5. Expanding the role of community health workers**

[If not mentioned earlier]. Do you think community health workers could be used to address these challenges? If so, how, in what capacities?

Probes:

- Encourage retrospective analysis of previous experiences with community health workers
- Encourage a reflection on specific challenges to, and opportunities for, the expansion of village health volunteers (VHVs), including policy and operational challenges
- Discuss specific issues related to financing and sustainability, integration in the wider health sector, and motivation

**6. Feasibility of rapid diagnostic tests and digital technologies for community-based interventions**

As part of this project, we envisage the introduction of low-cost, rapid tests and digital tools which can be used to diagnose non-malarial diseases and thus improve the management of non-malarial febrile illness in the communities. Can we discuss potential challenges and opportunities?

Probes:

- Direct the discussion around the key issues of sustainability, usability, procurement, acceptability, feasibility and integration into the wider health system
- Encourage a reflection on past experiences with rapid diagnostic tests

**7. Conclusions**

Thank you very much. Would you have anything do add? Do you have any questions? Would you have any suggestions about specific areas of investigation we should consider in future interviews? Could you name other informants we should talk to?
